# The patient experience with treatment and self-management (PETS) questionnaire: translation and cultural adaption of the Norwegian version

**DOI:** 10.1186/s12874-018-0612-9

**Published:** 2018-11-21

**Authors:** Anne Marie Lunde Husebø, Ingvild Margreta Morken, Kristina Sundt Eriksen, Oda Karin Nordfonn

**Affiliations:** 1 Department of Gastroenterological Surgery, Stavanger University Hospital, 4019 Stavanger, Norway; 2 Faculty of Health Sciences, University of Stavanger, 4036 Stavanger, Norway; 3 Department of Cardiology, Stavanger University Hospital, 4019 Stavanger, Norway

**Keywords:** Chronic illness, Cognitive interviewing, Colorectal cancer, Cross-cultural translation, Heart failure, Noncommunicable diseases, Patient-reported measure, PETS, Treatment burden

## Abstract

**Background:**

Noncommunicable diseases represents long term medical conditions, which often puts the patients under enormous demands when following treatment, exposing them to experiencing treatment burden. The Patient Experience with Treatment and Self-Management (PETS) questionnaire was developed as a patient-reported measure to identify treatment burden of chronic illness, using modern measurement theory and tested in a variety of settings. Developed in English, this set of measures had not been previously translated into Norwegian. The objective of this study was to develop a Norwegian version of the PETS and to pretest the translated measures through a cognitive debriefing methodology.

**Methods:**

A rigorous translation approach was applied, guided by Functional Assessment of Chronic Illness Therapy methodology. Bilingual teams from Norway and the United States reviewed the translation to develop a provisional version, which was evaluated for test content validity with cognitive interviews by probing 12 native Norwegian patients with noncommunicable diseases. The interviews applied both concurrent and retrospective verbal probing techniques, guided by a question route. Audio-recorded interviews were transcribed verbatim and analysed using systematic text condensation.

**Results:**

Assessment of translatability identified the need for cultural adaptation on several core words, balanced with the need to keep close to the original literal meaning. Seven patients with colorectal cancer and five patients with heart failure participated in cognitive testing of the Norwegian version of the PETS. The analytical process of the cognitive interviews identified two emergent main themes, ‘comprehension and readability’ and ‘relevance of the PETS’, with seven corresponding subthemes. Most items, response options and instructions were well understood by the patients. Revisions were made concerning cultural relevance.

**Conclusions:**

PETS items were semantically equivalent to the original. The patients with colorectal cancer and heart failure were able to comprehend the PETS and found it to express their experience with treatment burden in chronic illness. Future work will focus on psychometric construct validation and reliability testing of the PETS.

## Background

People with noncommunicable diseases (NCD) have chronic diseases, which are long lasting and progress slowly. Two of the main types of NCD diseases, cancer and cardiovascular disease, are demanding medical conditions and accounts for most NCD deaths [1]. People with cancer or cardiovascular disease may be especially vulnerable to treatment burden; many are required to engage in a complex variety of medical and self-management activities and some perceive the cognitive demands of adhering to their management regimen as onerous and difficult to meet [2, 3]. Thus, these patients are often required to seek care from a variety of providers, which can lead to lack of continuity and coordination between different levels of care [4, 5]. Treatment burden may be described as the *extra work*, i.e., self-care and self-monitoring, managing therapeutic regimens, organizing doctors’ visits, and managing transitions from hospitalization to outpatient treatment, that are delegated to the patients by health professionals [6, 7]. Some of the most profound consequences of treatment burden among patients include impaired health and well-being [8, 9], non-adherence to treatment [7], and high rates of re-hospitalization and emergencies [10].

Due to limited means of assessing patients’ ability to integrate complex care into their lives, a generic questionnaire to measure treatment burden; The Patient Experience with Treatment and Self-Management (PETS) was developed by Eton et al.from extensive conceptual work using qualitative data from patients with chronic disease [11–13]. This version of the PETS is the first to be translated into Norwegian.

If measures are to be used across cultures; the items must not only be translated well linguistically but also be adapted culturally in order to maintain the content validity of the instrument across cultures [14]. There is a need for patient reported measures to identify treatment burden in Norwegian NCD patients. Target NCD populations for pretesting in cognitive interviews (CI) in the present study include colorectal cancer (CRC) survivors and heart failure patients. This study aims to investigate the validity of a Norwegian culturally adapted version of the PETS as proposed by the Functional Assessment of Chronic Illness Therapy (FACIT) translation guidelines [15] and pretested by examining conceptual and cultural validity of concepts, items and semantic aspects in CIs with a sample of heart failure patients and CRC survivors.

## Methods

The PETS is a generic patient-reported measure originally developed in the USA and assesses aspects of treatment burden in patients experiencing chronic or long-term illness [13]. It was designed to assess 12 domains, which with its corresponding sub-scales covers medical information, medications, medical appointments, monitoring health, diet, exercise or physical therapy, medical equipment, relationship with others, medical and health care expenses, difficulties with health care services, role and social activity limitations, and physical and mental fatigue, resulting in a 60-item version [13]. The PETS has been used in prospective studies among chronically ill patients in the USA [16]. This version of the PETS is the first to be translated into Norwegian.

**Fig. 1:**
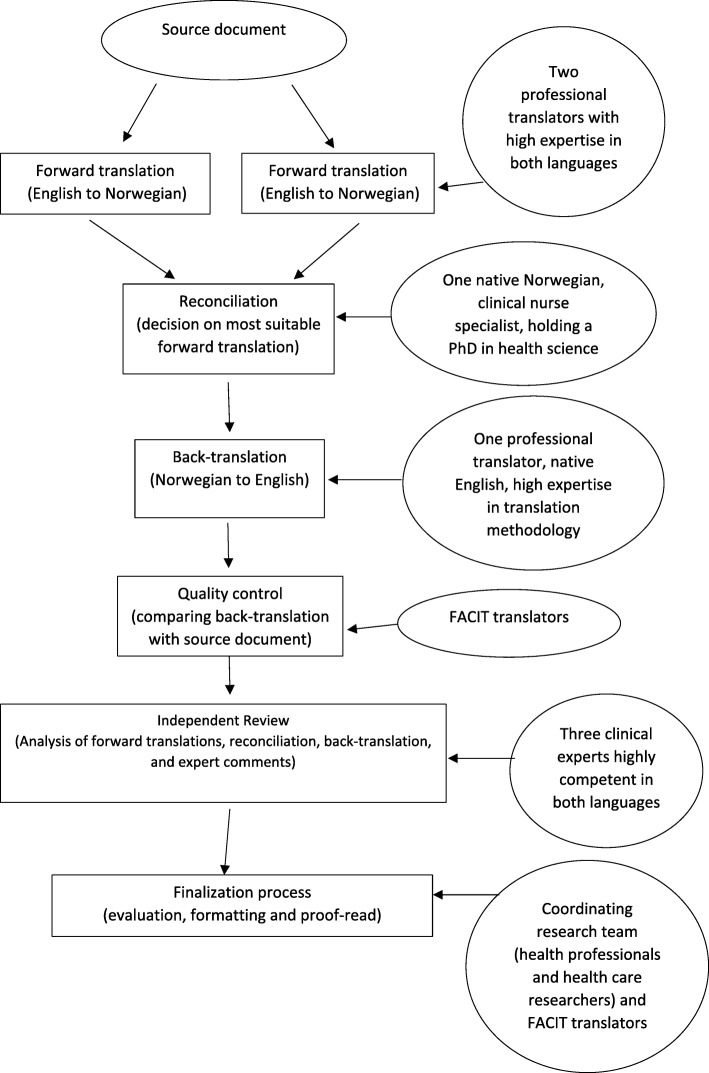
Translation process

The questionnaire was exposed to a rigorous translation approach guided by the FACIT translation guidelines [15], as recommended by the PETS developers. To reach equivalence between instruments, rigorous translation processes are required. We set out to perform forward translation, reconciliation review, back-translation, developer review, expert panel review and coordinating team evaluation (see Fig. 1), followed by cognitive interviews (CI) [15–17]. For initial testing of the Norwegian version of the PETS in CIs, a sample of 12 patients was recruited from two outpatient clinics at a university hospital in Western Norway. Inclusion criteria consisted of (1) age 18 years or older; (2) diagnosis of either chronic heart failure or CRC; and (3) being able to speak, read and write Norwegian. Participants were not eligible if they were diagnosed with terminal illness or with a cognitive deficiency.

### Translation process

Translation of the PETS was initiated with a forward translation from English to Norwegian that aimed to avoid literal translation and capture the item’s true meaning suitable for patients at a moderate education level. Two separate forward translations of the PETS were carried out individually by two native Norwegian professional translators, paired with two proof readers. These individuals were all educated in translation methodology, with 6–15 years of working experience as translators, including in the field of health research.

The two forward-translated versions were subjected to a reconciliation process carried out by a native Norwegian health professional holding a PhD in health science, who had extensive knowledge of the English language. This translation step focused on resolving discrepancies between the two Norwegian versions and providing alternative solutions to wording or content when considered necessary. The person performing the reconciliation was blinded to the source document.

The back-translation of the reconciled Norwegian version was performed by a native English-speaking professional translator who was fluent in the Norwegian language and had working experience as a health professional. The translator was blinded to the two forward-translated Norwegian versions and the source document and was instructed to use simple language and focus on literal meanings. A quality control of item meanings in the back-translated version was performed by the FACIT team by comparing the back-translated version with the source document, identifying equivalence and consistency between the source document and the target language version.

The next step of the translation process involved an expert panel of six people: two patients experienced with treatment of heart failure or CRC; two registered nurses (RN) experienced with nursing care to CRC survivors or heart failure patients; and two health science researchers experienced with nursing care of chronically ill patients (AMLH, OKN). The members of the expert panel independently analysed the Norwegian version of the PETS, using the reconciled version, the back-translated version, and comments made by linguistic experts at FACIT.org, to decide on the best Norwegian translation for each item. Item adjustments were documented by each reviewer in an item history sample page. After the experts’ comments had been carefully reviewed by the first and second authors accompanied by the FACIT team and the developers, a 60-item Norwegian version of the PETS was formatted and proof-read, finalizing the translation process.

### Cognitive interviews

The Norwegian version of the survey was pre-tested by use of CIs, a technique deriving from the cognitive question-answereing process of Tourangeau [18] to study the cognitive processes that respondents use to answer survey questions. In this study, Tourangeaus’ [18] process was used to guide the interviews, and to support pre-limenary analysis. According to Tourangeau, the respondent move through four different stages of 1) comprehension; 2) retrieving of necessary information, 3) jugdment of needed information, and finally 4) responding to the question [18]. These processes may be conscious or automatic, depending on the questions asked. By applying cognitive interviewing techniques one can prompt the respondents to reveal information that provides clues as to the types of processes respondents have used [18]. Also, during systemic debriefing of respondents, any flaws in words or phrases are checked. If needed, those words or phrases are further modified to establish the final version of the questionnaire [19].

Pretesting of the questionnaire through CI with the target population is vital as part of to achieving test content validity [17, 20]. Cognitive interviewing was done between drafting and administration of the Norwegian version of the PETS, aiming to initiate examination of validity from the intended test users’ perspectives. The Standards for Educational and Psychological Testing [20] states that *“Validation involves careful attention to possible distortions in meaning arising from inadequate representation of the construct and also to aspects of measurement, such as test format, administration conditions, or language level, that may materially limit or qualify the interpretation of test scores for various groups of test takers”* (p. 13). In this study, the CI’s were employed to establish evidence of test content (i.e. wording, item format, response tasks, administration and scoring), and preliminary evidence of BoT constructs, as measured by the Norwegian PETS. Also, CIs may identify items exposed to measurement error [17]. We anticipated that evidence of test content would address item meaning clarity, relevance and adequate coverage of BoT constructs.

Eligible patients were identified from patient lists at the outpatient clinics. Following a clinic appointment, the patients received face-to-face verbal and written information about the study from a clinic nurse, who then referred the patient to the researchers (AMLH, OKN) for consent and scheduling. After giving their written consent to participate, the participants received the questionnaire to complete at home, which allowed for replication of a trial setting [22].

The interviews were carried out by telephone, led by the first (AMLH) and the last author (OKN). Eleven interviews were audio-recorded. Written notes documented one interview with a cancer survivor. The interviews applied both concurrent and retrospective verbal probing techniques, a specialized questioning technique were the interviewer first ask the survey question and secondly probe an additional question to clarify comprehension, recall, decisions and judgement and response processes [17]. This technique focused on items that might be in danger of response error, as identified in the translation process. A question route based on Tourangeaus’ [18] cognitive question-answering process guided the interviews; including scripted probes (see Table 1). In addition, the interviewer used probes developed spontaneously during the interview. Probing was used to ask the patient to explain each item in his or her own words, identify deficiencies with the questions, and make judgements on PETS relevance in a Norwegian chronic illness context.

**Table 1:** Question route for cognitive interviews

Question	Category of cognitive probes
What does the term…mean to you?	Comprehension/Interpretation/Judgement probes
What do you understand by…?	
How did you arrive at that answer?	
Was it easy or hard to answer?	General probe
I noticed that you hesitated - tell me what you were thinking	
Do you have any closing remarks? (Debriefing)	

### Ethical considerations

Permission from the developer to translate and validate the PETS from English to Norwegian language for use in Norwegian health services context was obtained before initiating the adaption process. All the steps of the translation and validation process were made available to the Mayo Clinic developers, who approved the back-translated version and the final Norwegian version of the questionnaire.

The interview study was approved by the National Committee for Research Ethics in the Social Sciences and the Humanities (NESH) (Nos. 2017/284 and 2017/75) and by the university hospital research ethics board. Participation in the CIs was based on verbal and written informed consent. The participants received verbal information and an information letter from a nurse at the outpatient clinic. Further information was provided by the researchers (AMLH, OKN), who included the patients in the study prior to the CI. The participants were informed that they could withdraw their participation at any time. Audio-recordings and transcripts were anonymized and stored on the university hospital’s secure research server.

### Data analysis

The audio recordings were transcribed by two of the authors (AMLH, OKN). As preparation for the data analysis, patient statements were anonymized, checked for accuracy and incorporated into the item history sample page by AMLH and OKN. The analysis was carried out following Malterud’s [21] 4-step systematic text condensation (STC). STC is a qualitative analysis procedure inspired by Giorgi’s psychological phenomenological analysis aiming for a thematic analysis of meaning and content of data across cases [21]. In step 1, preliminary themes associated with Tourangeau’s [18] question-answering process were identified. In addition, themes of cultural and contextual appropriateness were added. This step was followed by identification and coding of meaning units (participants’ statements). In step 3, the meaning units were organized into seven sub-categories. In step 4, two main categories were identified.

## Results

### Translating the PETS from English to Norwegian

Challenges reported by the translators during the translation processes involved both linguistic and cultural concerns. During the reconciliation process and the developers’ quality control of the back-translated version, linguistic inconsistency with the source version was detected for nine items, requiring further translation and back-translation. The expert panel provided comments on 18 of 60 items, mainly involving minor grammatical issues, which did not raise any questions concerning mismatch between the source and the back-translated version. Assessment of translatability (i.e., reconciliation, expert panel, quality control) identified the need for cultural adaptations of core words. For instance, the word *medical appointments* was initially translated into the more generic Norwegian term for ‘appointments’, not particular to a health care service. The developers’ quality control suggested that to avoid confusion among respondents as to what kind of appointments this term concerned, *medical appointments* should be used, and this issue was evaluated during testing. In a Norwegian health care context, the concept of *health care providers* translates to both individuals and institutions, and a more literal Norwegian word pertaining to an individual level was chosen. It is also not appropriate to say *my* health care provider in Norwegian, thus ‘*my’* was excluded. Concerning the term *health care needs*, there is not an equivalent term in Norwegian, so we changed this to *need for health care services* as referring to the need for medical service. Two of the experts objected to the use of the Norwegian wording for *to stay healthy*, which in Norwegian can be interpreted as *to be cured*. The experts brought up that a chronic illness cannot be cured and suggested using *optimal functioning* instead. The quality control team found that this change altered the literal meaning of the item, and the original wording was kept for further evaluation. An item concerning health insurance coverage was recommended to be removed by one of the translators in the Norwegian version of the PETS due to extensive differences in health care coverage between the USA and Norway. The item was kept for further evaluation in CIs.

### Participants of the cognitive interviews

Twelve participants took part in the CIs, including seven CRC survivors and five patients with heart failure. The participants consisted of six men and six women, with an average age of 62 years (range 45–72) for the cancer survivors and 58 years (range 46–68) for the heart failure patients. All of the cancer survivors were diagnosed with rectal cancer (C20) and were classified into Duke cancer stages A-C. The patients with heart failure were diagnosed with either New York Heart Association (NYHA) Class II or III. The median time since diagnosis was 2 years for both patient groups (range 1–8 years). Eight of twelve participants reported on comorbidities. The median time of the recorded interviews was 32 min (range: 8–48 min).

### Themes

The analytical process (presented in Table 2) identified two main themes: ‘comprehension and readability’ and ‘relevance of the PETS’. The themes are illustrated by seven subthemes, and participants’ quotations were added to give meaning to the text.

**Table 2:** The analytical process of the cognitive interviews

Meaning units (a sample)	Sub-categories	Main categories
As far as I understood all the questions, they concern the experiences that people make in connection with visits to hospitals and health care, medicine use, and their experiences at the hospital, both with humans and medication, which are very important	Comprehension of item meaning	Comprehension and readability
It’s a medical appointment because you’re sick, right? And to deal with your illness, as well. I think it was a very relevant word to use, and a straightforward way to write it.	Comprehension of concepts	
And, where it says ‘diet’, I have basically written ‘no’ because I have never had a conversation with anyone about my diet because it has not been a problem	Retrieval of answers	
[Reads out loud] Organize -make appointments - keep track of - meet for appointments…they are much the same!	Survey layout and scope	
When it comes to doctors, I’ve met with different doctors each time, right? I don’t think I’ve met the same doctor twice, and when it comes to other personnel, it’s really the same (…) I think it’s very difficult, really… when you see a different physician every time you go in	Relevant to the patient groups	Relevance of the PETS
I do not know about others, but I think in this country, it [the health care system] is amazing! We really don’t pay anything, or at least I don’t. I pay the deductible of 2000 [Norwegian kroner]-and-something a year, afterwards everything is free	Cultural acceptance	
For me, much of this is not relevant because I have come so far in my illness	Timing of a treatment burden survey	
I do not feel it is relevant for my part. What I have is a long-term illness, and 4 weeks in this context, it is really nothing because it is there all the time	The last four weeks	Problematic recall time
The answer option “Does not apply to me”… I think it seems too easy to choose this answer Here, I missed the answer option “neither easy nor difficult” like for the other questions	Response alternatives	

### Theme 1: Comprehension and readability

This theme comprised the participants’ evaluation of the scope and layout of the PETS and their comprehension of the intended meanings and concept definitions of the items. The participants also explained how they reached appropriate answers to the questions.

#### Subtheme 1.1: Layout and scope of the PETS

The questionnaire was in general well received by the participants. Most of the participants found the number of items and scope to be appropriate and the layout to be comprehensible. A few respondents claimed that they would have to go back and read the instructions and questions again to gain an increased understanding. A small minority of participants found the PETS to be too long, with homonymous questions and a high level of detail. They started off with good intentions but ended up paying less attention to their responses in order to get it completed. One of the participants who was experienced in creating surveys had the following objections to the survey:

*‘I’m not a statistician, but most likely this is too detailed. I know that as an expert you become too detailed and that those you ask do not have the same detailed knowledge and that a lot of it becomes very similar. You start with good intentions and then it’s one page up and one page down... and so you think ... no, I won’t be bothered. So, I would have had fewer questions. Think of this as a starting point: how little can we include…because it often tends to be like this ... side up and side down. What is the minimum of what you want to know?’* (Cancer survivor).

Three of the PETS’ sub-scales begin with a screening question, where the participants can answer either *yes* or *no* before responding to the sub-scales’ items. If their answer is *no*, they can move on to the next sub-scale, leaving the items unanswered. This baffled some of the participants, claiming that they felt uncertain whether they had answered the question correctly.

#### Subtheme 1.2: Comprehension of item meaning

The participants expressed little difficulty in grasping the survey’s intended meaning and in general knew how to answer, as shown in this quotation:

*‘As far as I understood all the questions, they concern the experiences that people make in connection with visits to hospitals and health care, medicine use, and their experiences at the hospital, both with humans and medication, which are very important. I think the questions were all right. Now, I am in one situation and there are probably a thousand variations…, but I think it [the questionnaire] covered a lot.’* (Cancer survivor).

Among items that resulted in ambiguous responses from participants were items on whether they had received advice from health personnel on diet or exercise and physical therapy. Some participants found these items more difficult to answer because they could not relate to the situation of getting advice on health behaviours, while others had received recommendations and found the item meaningful. One of the participants explained:

*‘I thought it meant if you had received advice. I did not get any particular advice [on diet], but I got information about what to keep away from. In any case, this was not a question I found difficult to respond to*.’ (Cancer survivor).

#### Subtheme 1.3: Comprehension of concepts

The Norwegian wording and meaning of the concepts discussed by the expert panel as potentially unclear to patients were particularly mentioned in the interviews. Most concepts covered by the PETS were perceived by the participants as easy to understand, and they found them meaningful and relevant in a chronic illness context. A central target concept of the PETS is ‘self-management’, and the Norwegian translation of ‘self-management’ (egenhåndtering) engaged the participants of both diagnosis groups. When asked to think aloud, one of the cancer survivors responded:

*‘I think this means how easy it is to control your agenda related to illness and health ... how well do you manage to juggle everything ... and then it also makes sense in relation to physical and mental fatigue.’* (Cancer survivor).

Some viewed the concept of self-management as fabricated during the translation process and therefore not well integrated in the Norwegian language:

*‘I think I have heard it before, but I think it is rarely used.’* (Cancer survivor).

Others suggested it be changed to the more familiar word ‘self-care’.

Participants thought of ‘health professionals’ mainly as doctors, including their general practitioner (GP) and nurses, while ‘medical appointments’ was explained by several participants as appointments referred to them by their GP or by a specialist at the hospital. In Norwegian, the word ‘appointment’ also translates as ‘agreement’, which confused some of the participants, referring to the agreements they had made with their physician regarding medication and other treatments:

*‘For my part, it’s the heart failure department that is managing my medicine right now. So, I understood it like that.’* (Heart failure patient).

*‘If the doctor says that I have to take this medicine, then I have to take it. You don’t go beyond what the doctor tells you.’* (Cancer survivor).

#### Subtheme 1.4: Retrieval of answers

The interviews revealed that despite a retrospective recall period of ‘the last four weeks’, participants used both prospective and retrospective associations to reach their answers to the questions. Prospectively, the participants related their answers to what could happen in future encounters with the health care services. One participant explained how he reached the response alternative ‘strongly agree’ to the assertion ‘I have to go to too many specialists for my health problems’:

*‘I was thinking that I’m inside this system, so I am being sent from one medical exam to another.’* (Heart failure patient).

To the question ‘With regard to your health needs, how easy or difficult has it been for you in the last 4 weeks to make or attend appointments?’ one cancer survivor replied:

*‘In my opinion, I will answer “easy” because if you are referred to a specialist, you will meet at the time you have been given, and if I am late to the appointment, I will call them and let them know.’* (Cancer survivor).

Retrospective association was used by respondents when they thought back on particular encounters with the health care services they had experienced in the past. In the following quotation, a patient with heart failure explained the process of deciding on an answer:

*‘Here, I have not filled in anything, just answered ‘no’ because there has never been anyone who has talked to me about any diet or mentioned something of the kind. The only thing I’ve been to is lectures when I was in the hospital…about heart disease and diet. No one has told me ‘you should not eat that’ or ‘you should eat more of this’.’* (Heart failure patient).

### Theme 2: Relevance of the PETS

When participants expressed their opinions of the relevance of the questionnaire, they related it to their diagnosis and medical history and whether the questionnaire seemed appropriate in a Norwegian health care setting. They also had thoughts concerning the right time to respond to a treatment burden survey, in light of the time since diagnosis and primary treatment.

#### Subtheme 2.1: Relevance to the patient groups

The relevance, content and themes of the PETS became obvious from the participants’ comments about particular encounters with the health care services and from responses to the interviewers’ probing regarding the relevance of specific items. The majority of the sub-scales were relevant. Most participants could relate to how following up on treatment regimens and how their meetings with health professionals affected their daily lives and functionality, as well as their relationships with others. In response to the interviewers’ question of the relevance of the sub-scale ‘Difficulties with the health care services’, several of the respondents had experienced a long wait for specialist appointments, as illustrated in this quote:

*‘I had to wait half a year to get an appointment with him [the specialist], which lasted for 5 minutes… and then I had to wait 3 months before I got an appointment in Bergen. Then, I was there for 3 minutes, as well as a 24-hour wait, and then it was home again, and wait for 3 more months before I could go back. There is a lot of waiting and waiting and waiting… which annoys me.’* (Heart failure patient).

Others expressed the relevance of the PETS because they recognized the challenges raised in questions concerning organizing medications, maintaining a diet or exercise routine, relationships with others, experiencing fatigue and being socially restrained by the treatment burden.

To the participants in both diagnosis groups, relevance also included a discussion of the shortcomings of the survey and suggestions for the inclusion of new perspectives. For some of the cancer survivors, the treatment had resulted in an altered body image, which caused emotional distress, particularly in relation to others. This issue was brought forward by one of the participants, who talked about how the stoma affected self-image and quality of life:

*‘It was the stoma that was the hardest and most important issue ... the hardest to accept and which was the best thing to get rid of. That was something that weighed down on me every day. That theme should have been included in a separate section [of the PETS].’* (Cancer survivor).

One participant discussed with the interviewer the different listed moods in the subscale ‘Physical and mental exhaustion’, pointing out that they did not cover emotions of despair relating to the burden of heart failure:

Heart failure patient: *‘Like “mad”…I’m never angry, I’m not. So I’ve answered “never”. I’ve been sad and worried, so I’ve answered “often”. And I’ve often been depressed and worn out…and sometimes frustrated too…so have answered “often” and “sometimes”.’*

Interviewer: *‘Yes, but what about “being sad”, is that what you think is missing?’*

Heart failure patient: *‘Yes, being sad, and thinking “why all this…”.’*

#### Subtheme 2.2: Cultural relevance

Participants’ expressions related to the cultural relevance of the survey were largely based on their opinions of the sub-scale ‘Medical and health care expenses’. Here, they were asked to answer questions about how easy or difficult it had been to pay for their own medicines, buy healthy food, etc. Many respondents had opposing opinions on the relevance of this sub-scale and whether it applied to the Norwegian social security system of health care services refund. They expressed being in a fortunate position due to the refund system, claiming that the expenses were few and that they thought most people could handle them:

*‘I do not know about others, but I think in this country, it [the health care system] is amazing! We really don’t pay anything, or at least I don’t. I pay the deductible of 2000 [Norwegian kroner]-and-something a year, afterwards everything is free.’* (Heart failure patient).

The same applied for items about the availability of healthy foods and the economic burden of buying them:

*‘Is this really a relevant question? I would have answered ‘no’…I think most people will manage to find healthy food… I think everyone can handle it.’* (Cancer survivor).

In contrast, other respondents raised the issue that some medicines prescribed by specialists are not covered by the social security system and that healthy foods like vegetables are more expensive than junk food and soft drinks, proving the relevance of the items.

#### Subtheme 2.3: Relevant timing of surveys on treatment burden

Both patient groups had concerns about whether it was relevant to inquire about patient experiences of treatment burden years after the time of diagnosis and primary treatment. These concerns were evident for the cancer survivors in particular and among the participants who had lived with heart failure for many years, as expressed in these quotations:

*‘To me, a lot of this is not relevant because I have come so far in my illness, you see. But, if you give it to the patients early, I think a lot can be disclosed.’* (Heart failure patient).

*‘When it comes to the question on transport to and from the hospital, it depends on which stage of the illness you are in. If you had asked me four months ago I would have answered “very difficult”.’* (Cancer survivor).

Cancer survivors perceived the PETS items as more relevant if received closer to the end of primary cancer treatment, when they experienced the greatest treatment burden, as expressed by one participant:

*‘I think the questionnaire is comprehensible, but as I said, I think it is a bit too long ago since my treatment. To me, six months following hospital discharge would have been the right time to answer the questions because that was when I had the biggest problems.’* (Cancer survivor).

The participants also spoke of relevant timing in relation to the recall time of four weeks, which several of the participants in both diagnosis groups found hard to relate to. Thinking back in time was difficult and somewhat confusing because time had passed since the treatment intensity was at its highest, which was particularly applicable among cancer survivors, who expressed that they had moved on and that the treatment burden had diminished. One participant said the following in response to sub-scale items on advice about a healthy diet:

*‘I did get some information at the first check-up about things I should avoid due to my stoma. The ‘last four weeks’ means that it is not relevant to me anymore because I got the colon put back in.’* (Cancer survivor).

The recall time of four weeks was also described by the participants as falling short within the context of long-term illness, as it can be difficult to distinguish one period of time from another:

*‘I do not feel it is relevant for my part. What I have is a long-term illness, and 4 weeks in this context, it is really nothing…because it is there all the time.’* (Heart failure patient)

[Reads out load] *‘“The last four weeks”... From my experience, it is meant to make people respond based on their experiences from the last four weeks, but you are responding to the insight you have now, and it can be difficult to distinguish between, “Have I had this insight over the last four weeks, or have I had it for two months or six months?”’* (Cancer survivor).

## Discussion

This study described the process of translation and evaluation of the PETS by use of FACIT translation methodology [15], including cognitive interviews (CI). The final Norwegian version of the PETS contains 59 questions, divided into 12 domains, covering a wide range of topics relevant to the experienced burden of treatment in long-term illness.

The CI methodology aimed to understand how respondents comprehended and generated their answers to the PETS items and revealed issues concerning both semantic and conceptual appropriateness. In addition, the CIs allowed for non-cognitive defects in the questionnaire to be detected [17]. The CIs raised few concerns regarding structural or logical problems (e.g., unclear layout, erroneous skip-patterns) or about the questionnaire being too extensive and time-consuming. Only a few items were on the cusp of being too long (i.e., > 16 words). Sentences containing over 16 words may confuse the respondent in terms of the main message of the question [22]. Potential future skip-patterns regarding domains initiated by a Yes/No condition (PETS domains of ‘diet’, ‘exercise and physiotherapy’, and ‘medical equipment’) were detected. Skip-patterns, as reported by the participants, were due to a lack of experience with the subscale thematics during the recall period. Skip-patterns in survey development are used to avoid data entry errors and to enable a more efficient questionnaire administration [23] but can result in missing data. Eton et al. [13] found that skip-patterns from the aforementioned PETS subscales resulted in 46–61% missing data, and the scales had to be excluded from factor analysis.

Some problems regarding response formulating caused by the translation were identified, mainly concerning the fact that some English words have more than one meaning in Norwegian. This problem could cause biased answers in future surveys and stresses the need for testing in target-language populations [17]. In the current study, the word ‘appointment’ confused some of the participants because it also translates into the Norwegian word for ‘agreement’. In the source version of the PETS, ‘medical’ has been added to distinguish it from appointments other than those made with health professionals. In addition, survey respondents may be provided with the context of key ideas by adding clarifying instructions to sub-scales [22]. Some respondents detected structural problems with the Norwegian PETS, like similarity of wording between items. Structural or logical defects of surveys may cause confusion when responding, result in missing or biased information, and consequently cause bias correlations between variables, threatening the survey’s validity [17]. Repeated testing in CIs is recommended to optimize survey layout and minimize measurement errors [17].

The relevance of a BoT measurement for use among Norwegians living with long-term illness was established on several levels, indicating a proper fit for capturing patients’ experiences following treatment and the impact of treatment on daily life functioning and wellness. However, the translation process identified discrepancies related to different word choices and sentence order and raised questions regarding literal translation versus paraphrasing of terms. To allow for comparison of scores across cultures, and pending psychometric testing of validity, changing of words was kept to a minimum and restricted to issues of translation difficulties [15].

Discussions of the survey’s cultural relevance were largely based on opinions of the sub-scale ‘Medical and health care expenses’ brought forward both by the expert panel and during CIs. The PETS scale was developed for use in a health care system that is culturally very different from the Norwegian system and raises the question of whether queries on social security systems and of health care service refunds can be transferred between cultures. Brislin [24] refers to the process of decentering, which in some cases can be used to modify both the source instrument and the translation to obtain equivalence of meaning between them. Decentering during translation of materials from one language and culture to another aims at producing a translated version centred on the target population’s language and culture [24]. Due to ongoing testing of the source instrument and to allow for comparisons of scores across translated versions, decentring was not recommended in the process of translating the PETS.

### Strengths and limitations

One strong point of this study is that it followed a well-tested and rigid methodology for cross-cultural translation and validation of patient reported measures [15]. The parties involved in the translation process were knowledgeable and experienced within the field of survey development and adaption and held high positions within chronic illness research. The advantages of the chosen methodological approach of using telephone interviews included; a prior, personal contact with the respondents [25]; no travel inconveniences or expenses for the respondents [17]; and that the telephone may have granted the respondents with partial anonymity, which gave them the opportunity to speak more freely regarding their experiences with the health care services [26].

The respondent debriefing approach allowed the interviewer to control the exchange of information by probing and asking follow-up questions [27]. This approach provided a good method for testing the respondents’ comprehension of PETS concepts that were revealed as difficult to handle during translation and for verifying that questions were comprehended as intended on a semantic and conceptual level.

Testing of the Norwegian version of the PETS was limited to two NCD patient populations, which may question the generalisabiliy of the study. Never the less, the patient populations participating in this study may serve as proxies for other chronically ill patient populations. Thus, the PETS holds potential for use among individuals suffering from a range of chronic illness. In clinical settings, the use of patient reported measures such as this may facilitate appropriate care management and improved health outcomes for a growing population suffering from chronic illness. Additionally, BoT instruments can be used as a tool for quality improvement initiatives.

Although the CI technique aided the identification of problematic questions on all four stages of Tourangeau’s question-answering process [18], some limitations should be addressed. First, a risk of testing in CIs is that the judgements given are of a qualitative, subjective nature and may not affect all respondents in future surveys. Thus, the detected problems may be questioned as not “real”. However, validity may be judged based on how often the problem occurs across interviews and if they most likely will perform badly during field data collection and lead to biased data [28]. In our study, this was the case for the cultural relevance of the subscale ‘Medical and health care expenses’, which seemed problematic to several of our respondents. By listening to the voices of the target population and conferring with the survey developers, our translation team carefully reviewed all of the feedback and identified the most unclear and problematic item, which was deemed culturally irrelevant and removed from the Norwegian version. According to Tourangeau [18], change of context may affect the comprehension process, leading to a totally different reading of a survey item.

Secondly, while the literature conclude that telephone interviews may be a valuable and valid method for data collection, using telephone interviews versus face-to-face interviews is debatable. Respondents’ facial expressions or bodily cues were not visible to the interviewer, which could have added additional information to the participants’ responses [24].

As a third limitation, the possibility for investigatory bias in the analysis of the CI data cannot be ruled out. Nonetheless, the interviews were read by all authors to identify preliminary themes, and all authors were involved in coding and categorizing. Finally, this study did not confirm that the psychometric properties of the source document were preserved in the Norwegian version, so more research is required to further examine internal consistency reliability and construct validity of the Norwegian PETS.

## Conclusions

This study is the first to translate and qualitatively evaluate test content validity of a patient reported measure that will be clinically useful in assessing and addressing the detailed experience with treatment and self-management among two main NCD populations in Norway. As a translational study, it provides insight into cross-cultural questionnaire development methodology, aiming at providing a measurement that is useful and comprehensible to the target population.

Although the processes of translation and cognitive testing were time-consuming, costly and rigid, they proved to constitute a sound survey methodology that is appropriate to provide detailed information about the dimensions of BoT. The main challenge was related to the choice between semantic and linguistic equivalence with the source document vs. decentring, as the latter would result in changes that could alter the intended meanings of the items and make cross-cultural comparison difficult. The newly adapted version of the PETS has high semantic equivalence to the original survey and was well accepted by the target population. The Norwegian version of the PETS is now available for further studies to assess validity and reliability.

## Abbreviations

BoT: Burden of treatment
CI: Cognitive interview
CRC: Colorectal cancer
FACIT: Functional Assessment of Chronic Illness Therapy
NCD: Noncommunicable disease
PETS: Patient Experience with Treatment and Self-management

## References

[1] World Health Organization. Noncommunicable diseases and their risk factors. 2018. Available from: http://www.who.int/ncds/introduction/en/. Accessed 12 Nov 2018.

[2] Feltner C, Jones CD, Cenè CW, Zheng ZJ, Sueta CA, Coker-Schwimmer EJL (2014). Transitional care interventions to prevent readmissions for persons with heart failure: a systematic review and meta-analysis. Ann Intern Med.

[3] Jakobsson J, Idvall E, Kumlien C (2017). The lived experience of recovery during the first 6 months after colorectal cancer surgery. J Clin Nurs.

[4] Olano-Lizarraga M, Oroviogoicoechea C, Errasti-Ibarrondo B, Saracíbar-Razquin M (2016). The personal experience of living with chronic heart failure: a qualitative meta-synthesis of the literature. J Clin Nurs.

[5] Kotronoulas G, Papadopoulou C, Burns-Cunningham K, Simpson M, Maguire R (2017). A systematic review of the supportive care needs of people living with and beyond cancer of the colon and/or rectum. Eur J Oncol Nurs.

[6] Sav A, Kendall E, McMillan SS, Kelly F, Whitty JA, King MA, Wheeler AJ (2013). ‘You say treatment, I say hard work’: treatment burden among people with chronic illness and their careers in Australia. Health Soc Care Commun.

[7] May CR, Eton DT, Boehmer K, Gallacher K, Hunt K, MacDonald S (2014). Rethinking the patient: using burden of treatment theory to understand the changing dynamics of illness. BMC Health Serv Res.

[8] Tran VT, Montori VM, Eton DT, Baruch D, Falissard B, Ravaud P (2012). Development and description of measurement properties of an instrument to assess treatment burden among patients with multiple chronic conditions. BMC Med.

[9] Tran VT, Barnes C, Montori VM, Falissard B, Ravaud P (2015). Taxonomy of the burden of treatment: a multi-country web-based qualitative study of patients with chronic conditions. BMC Med.

[10] Schneider EB, Hyder O, Brooke BS, Efron J, Cameron JL, Edil BH (2012). Patient readmission and mortality after colorectal surgery for colon cancer: impact of length of stay relative to other clinical factors. J Am Coll Surg.

[11] Eton DT, Ramalho de Oliveira D, Egginton JS, Ridgeway JL, Odell L, May CR, Montori VM (2012). Building a measurement framework of burden of treatment in complex patients with chronic conditions: a qualitative study. Patient Relat Outcome Meas.

[12] Eton DT, Ridgeway JL, Egginton JS, Tiedje K, Linzer M, Boehm DH (2015). Finalizing a measurement framework for the burden of treatment in complex patients with chronic conditions. Patient Relat Outcome Measures.

[13] Eton DT, Yost KJ, Lai JS, Ridgeway JL, Egginton JS, Rosedahl JK (2017). Development and validation of the patient experience with treatment and self-management (PETS): a patient-reported measure of treatment burden. Qual Life Res.

[14] Beaton DE, Bombardier C, Guillemin F, Ferraz MB (2000). Guidelines for the process of cross-cultural adaptation of self-report measures. Spine (Phila Pa 1976).

[15] Eremenco SL, Cella D, Arnold BJ (2005). A comprehensive method for the translation and cross-cultural validation of health status questionnaires. Eval Health Prof.

[16] Rogers EA, Yost KJ, Rosedahl JK, Linzer M, Boehm DH, Thakur A (2017). Validating the patient experience with treatment and self-management (PETS), a patient-reported measure of treatment burden, in people with diabetes. Patient Relat Outcome Measures.

[17] Willis GB (1999). Cognitive interviewing: a “how to” guide.

[18] Tourangeau R (1984). Cognitive sciences and survey methods. Cognitive aspects of survey methodology: Building a bridge between disciplines.

[19] Collins D (2003). Pretesting survey instruments: an overview of cognitive methods. Qual Life Res.

[20] American Educational Research Association; American Psychological Assocoation; National Council of Measurement in Education. Joint Committee on Standards for Educational and Psychological Testing (US). Washington DC: American Educational Research Association; 2014.

[21] Malterud K (2012). Systematic text condensation: a strategy for qualitative analysis. Scand J Public Health.

[22] Brislin RW, Lonner WL, Berry JW (1986). The wording and trasnslation of research instruments. Field methods in cross-cultural research.

[23] Setia MS (2017). Methodology series module 8: designing questionnaires and clinical record forms. Indian J Dermatol.

[24] Brislin RW (1976). Comparative research methodology: cross-cultural studies. Int J Psychol.

[25] Polit DF, Beck CT. Nursing research: principles and methods. Philadelphia: Lippincott Williams & Wilkins; 2004.

[26] Sturges JE, Hanrahan KJ (2004). Comparing telephone and face-to-face qualitative interviewing: a research note. Qual Res.

[27] Liu Y, Hinds PS, Wang J, Correia H, Du S, Ding J (2013). Translation and linguistic validation of the pediatric patient-reported outcomes measurement information system measures into simplified Chinese using cognitive interviewing methodology. Cancer Nurs.

[28] Conrad FG, Blair J, Groves RM, Kalton G, Rao J, Schwarz N, Skinner C, Presser S (2004). Data quality in cognitive interviews: the case of verbal reports. Methods for testing and evaluating survey questionnaires.

